# From Dieulafoy’s Lesion to Gastrointestinal Stromal Tumor: A Case of Recurrent Upper Gastrointestinal Bleeding

**DOI:** 10.7759/cureus.113214

**Published:** 2026-07-23

**Authors:** Ala Hamid Ali Suliman, Aaliyah Kheruwala, Ahmad Barbour, Sara Eltayeb, Ahmed Khassouan

**Affiliations:** 1 Internal Medicine, Mohammed Bin Rashid University of Medicine and Health Sciences, Dubai, ARE; 2 Gastroenterology, Rashid Hospital, Dubai, ARE

**Keywords:** dieulafoy lesion, dieulafoy’s lesion, gastric gist, gastrointestinal stromal tumor, laparoscopic wedge resection, recurrent gastrointestinal bleeding, upper gastrointestinal bleeding, upper gi endoscopy

## Abstract

Dieulafoy’s lesion and gastrointestinal stromal tumors (GISTs) are uncommon causes of upper gastrointestinal (GI) bleeding. We report a rare case of recurrent upper GI bleeding in a 71-year-old male who initially presented in 2017 with hematemesis, melena, and hemodynamic instability. Upper GI endoscopy identified a fundal Dieulafoy’s lesion, which was successfully treated with endoscopic clipping. Seven years later, the patient re-presented with melena, fatigue, dizziness, and symptomatic anemia requiring blood transfusion. Repeat endoscopy demonstrated a 6-cm ulcerated submucosal mass in the gastric fundus. Endoscopic ultrasound showed a lesion arising from the fourth layer of the gastric wall, and fine-needle aspiration confirmed a low-grade mixed-type GIST. Computed tomography demonstrated a localized enhancing gastric mass without evidence of metastatic disease. Following multidisciplinary review, the patient underwent successful laparoscopic wedge resection. Histopathological examination confirmed a completely excised low-risk gastric GIST measuring 6.5 × 5.5 × 4.5 cm with a mitotic rate of 3 mitoses per 5 mm² and negative surgical margins. The postoperative course was uneventful. This case highlights the importance of reassessing patients with recurrent GI bleeding despite a previously identified bleeding source. The unusual temporal association between a Dieulafoy’s lesion and a subsequently diagnosed gastric GIST in the same anatomical region underscores the need for continued diagnostic vigilance and multidisciplinary management in atypical presentations of upper GI bleeding.

## Introduction

Gastrointestinal (GI) bleeding is a common yet potentially life-threatening medical emergency, caused by a variety of pathologies ranging from benign disorders like peptic ulcers to more complicated ones, including tumors and vascular abnormalities [1]. While most cases are attributable to common etiologies, uncommon causes such as Dieulafoy’s lesion and gastrointestinal stromal tumors (GISTs) present significant diagnostic challenges due to their variable and often atypical clinical presentations [2].

Dieulafoy’s lesion is a rare but significant cause of upper GI bleeding, caused by a large submucosal artery protruding through intact mucosa, typically in the stomach. Although it accounts for only 1-5% of upper GI bleeds, it can lead to severe, potentially life-threatening hemorrhage [1]. Diagnosis can be challenging due to intermittent bleeding and the possibility of missed lesions during initial endoscopic evaluation. Endoscopic identification and treatment may require careful evaluation and, in some cases, repeated procedures using therapeutic modalities such as clipping, injection therapy, or thermal coagulation [3]. GISTs are rare mesenchymal neoplasms arising from the interstitial cells of Cajal within the GI tract. They occur most frequently in the stomach (60-70%) and small intestine (25-35%). Although many GISTs are detected incidentally, approximately 70% of patients are symptomatic at diagnosis, commonly presenting with GI bleeding secondary to mucosal ulceration or tumor erosion. The liver and peritoneum are the most frequent sites of metastatic disease, while lymphatic spread is uncommon. Evaluation for metastatic or synchronous lesions is therefore an important component of staging and treatment planning [2,4].

While both Dieulafoy’s lesion and GISTs are independently uncommon causes of upper GI bleeding, their coexistence or sequential occurrence has rarely been described in the literature [2]. Current published literature includes only a small number of case reports describing GISTs associated with Dieulafoy-like vascular lesions or prominent tumor-associated vessels presenting with GI hemorrhage. These reports highlight the diagnostic challenges posed by these uncommon entities and emphasize the importance of maintaining a broad differential diagnosis in patients with recurrent bleeding [2,5-8]. Furthermore, previous studies have demonstrated that repeat esophagogastroduodenoscopy and colonoscopy may improve the diagnostic yield in patients with persistent or recurrent GI bleeding when initial investigations fail to identify the underlying source [5].

We report a unique case of a patient who initially presented with upper GI bleeding secondary to a Dieulafoy’s lesion and experienced recurrent bleeding seven years later due to a gastric GIST arising in the same anatomical region. This case highlights the importance of considering uncommon etiologies in patients with recurrent GI bleeding and emphasizes the value of repeated evaluation and multimodal diagnostic approaches when symptoms recur.

## Case presentation

A male with a history of hypertension and hyperlipidemia experienced two episodes of upper GI bleeding separated by seven years. His initial presentation occurred in 2017 with a Dieulafoy’s lesion, while his recurrent presentation in 2024, at the age of 71 years, led to the diagnosis of a gastric GIST.

**Figure 1:**
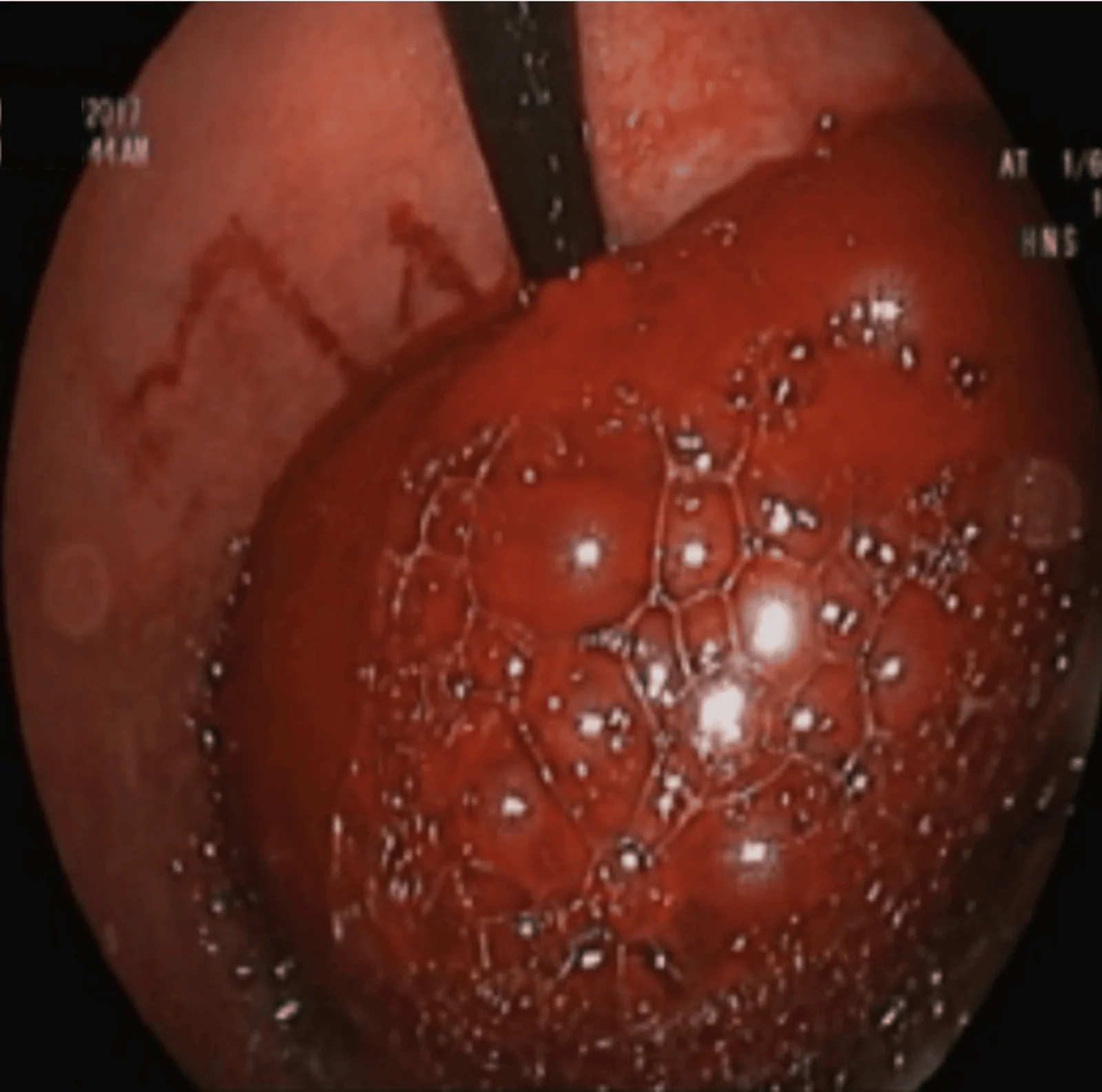
Large blood clot covering the fundus.

**Figure 2:**
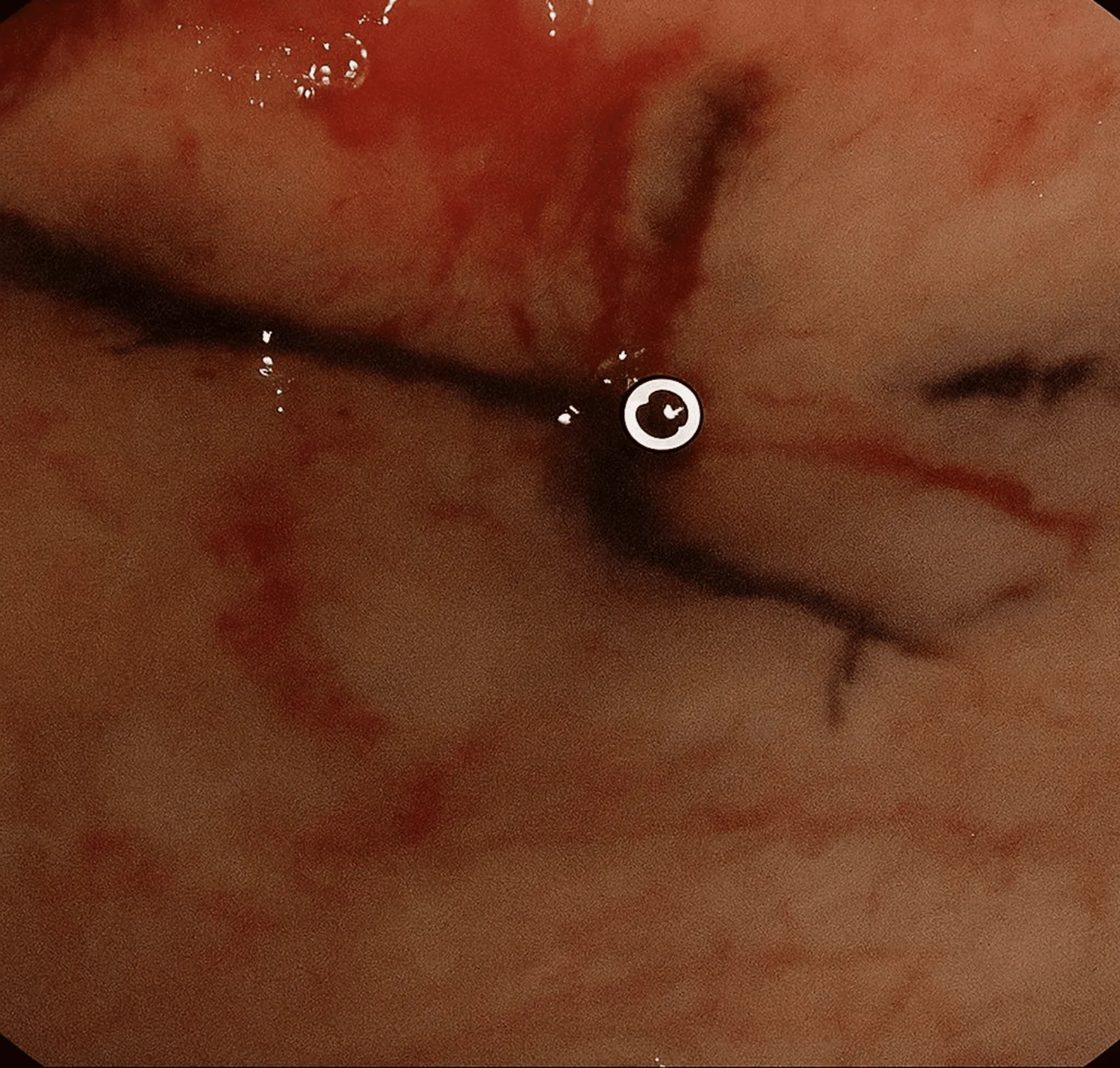
Dieulafoy's lesion in the fundus, hemoclip applied.

In 2017, he presented to the emergency department with hematemesis, melena, fatigue, and hemodynamic instability. Laboratory investigations revealed severe anemia with a hemoglobin level of 7.0 g/dL, requiring blood transfusion (Table 1). Upper GI endoscopy demonstrated a large adherent blood clot within the gastric fundus, obscuring visualization of the underlying lesion (Figure 1). Following clot removal, an isolated protruding vessel overlying normal-appearing mucosa was identified, consistent with a Dieulafoy lesion (Figure 2). Endoscopic hemostasis was successfully achieved with hemoclip placement. The patient remained clinically stable following the procedure and was discharged home.

**Table 1:** Relevant laboratory findings during the two presentations with upper gastrointestinal bleeding. MCV: mean corpuscular volume; MCH: mean corpuscular hemoglobin; RDW: red cell distribution width; INR: international normalized ratio.

Parameter	2017 presentation	2024 presentation	Reference range	Interpretation
Hemoglobin (g/dL)	7.0	7.8	13.0-17.0	Severe anemia
Hematocrit (%)	21.2	21.9	40-50	Decreased
RBC count (×10¹²/L)	2.23	2.34	4.50-5.90	Decreased
MCV (fL)	95.2	93.8	80.0-100.0	Normocytic
MCH (pg)	31.5	33.5	27.0-33.0	Normal; mildly elevated in 2024
RDW (%)	14.1	13.3	11.5-14.5	Within normal limits
White blood cell count (×10⁹/L)	16.0	9.9	4.0-11.0	Elevated in 2017; normal in 2024
Platelet count (×10⁹/L)	110	196	150-400	Mild thrombocytopenia in 2017; normal in 2024
Urea (mg/dL)	23	18	15-40	Within normal limits
Creatinine (mg/dL)	0.50	0.83	0.70-1.30	Normal renal function
INR	1.25	1.14	0.80-1.20	Mildly elevated in 2017; normal in 2024

In 2024, the patient re-presented with generalized fatigue, exertional dyspnea, dizziness, near-syncope, and melena of four days’ duration. He denied the use of nonsteroidal anti-inflammatory drugs, antiplatelet agents, or anticoagulants. On examination, he appeared pale and demonstrated orthostatic hypotension. Laboratory investigations again revealed significant anemia with a hemoglobin level of 7.8 g/dL, necessitating transfusion of two units of packed red blood cells.

**Figure 3:**
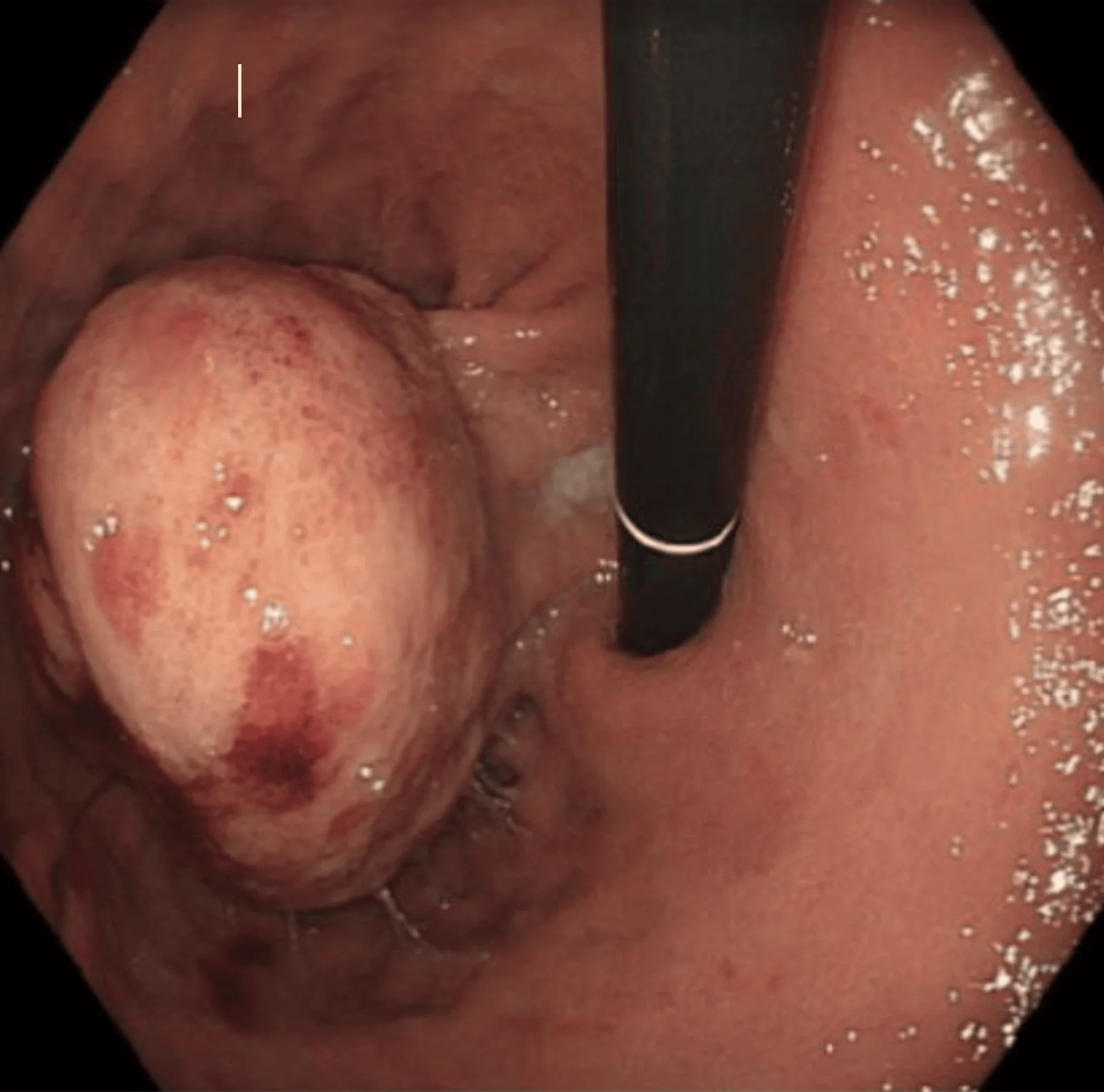
A 6 cm submucosal mass in the fundus, more than 5 cm away from the cardia.

Upper GI endoscopy revealed a large ulcerated submucosal mass measuring approximately 6 cm in the gastric fundus, located more than 5 cm distal to the cardia (Figure 3). A small clean-based ulcer overlying the lesion was identified without evidence of active bleeding. No endoscopic hemostatic intervention was required, and the patient was managed conservatively with intravenous pantoprazole.

Endoscopic ultrasound demonstrated a mass arising from the fourth layer of the gastric wall. Fine-needle aspiration confirmed a low-grade mixed-type GIST. Contrast-enhanced computed tomography of the abdomen demonstrated an enhancing endoluminal gastric mass without evidence of metastatic disease.

**Figure 4:**
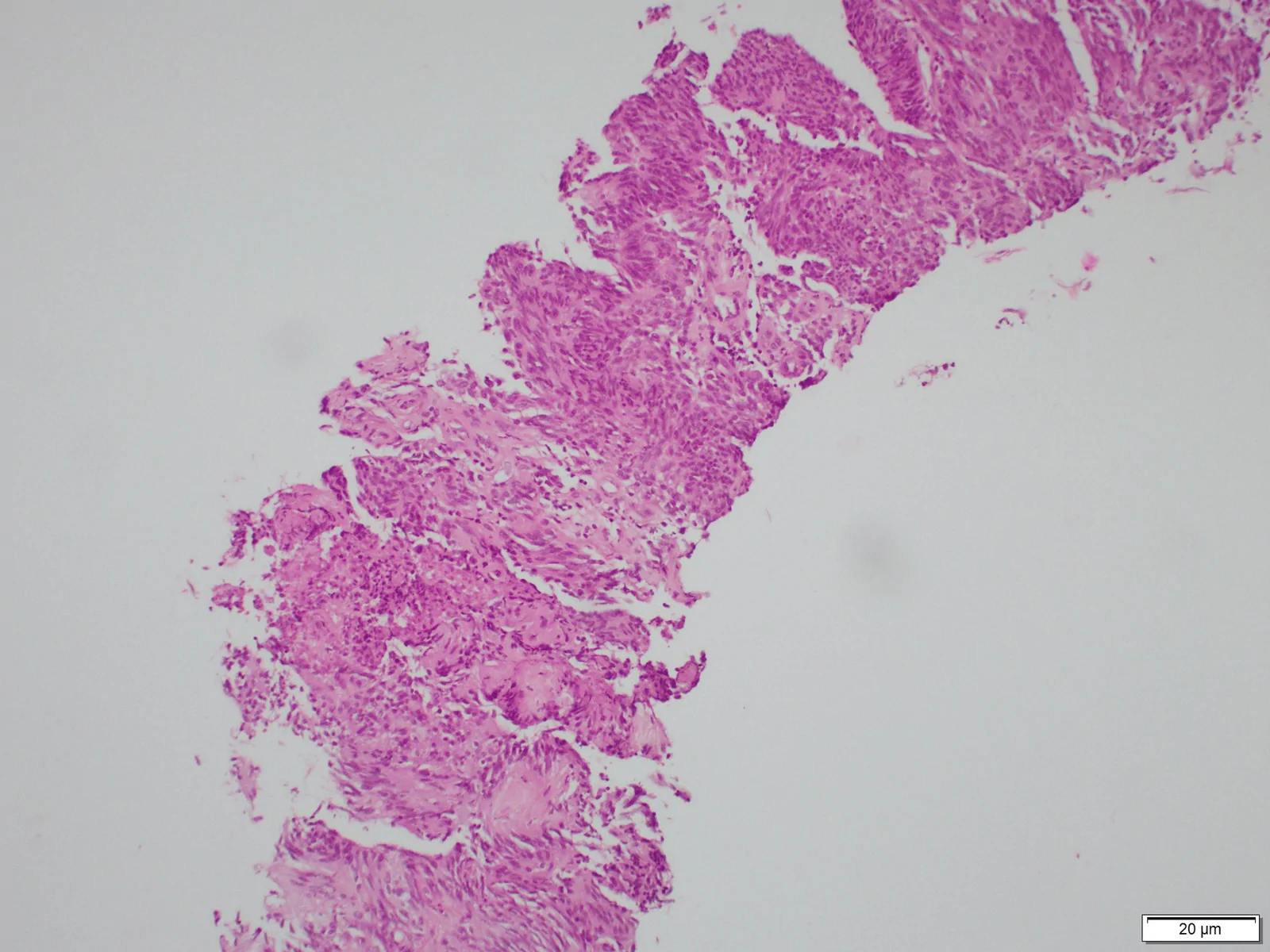
Low-power view (4×) showing a spindle cell lesion.

**Figure 5:**
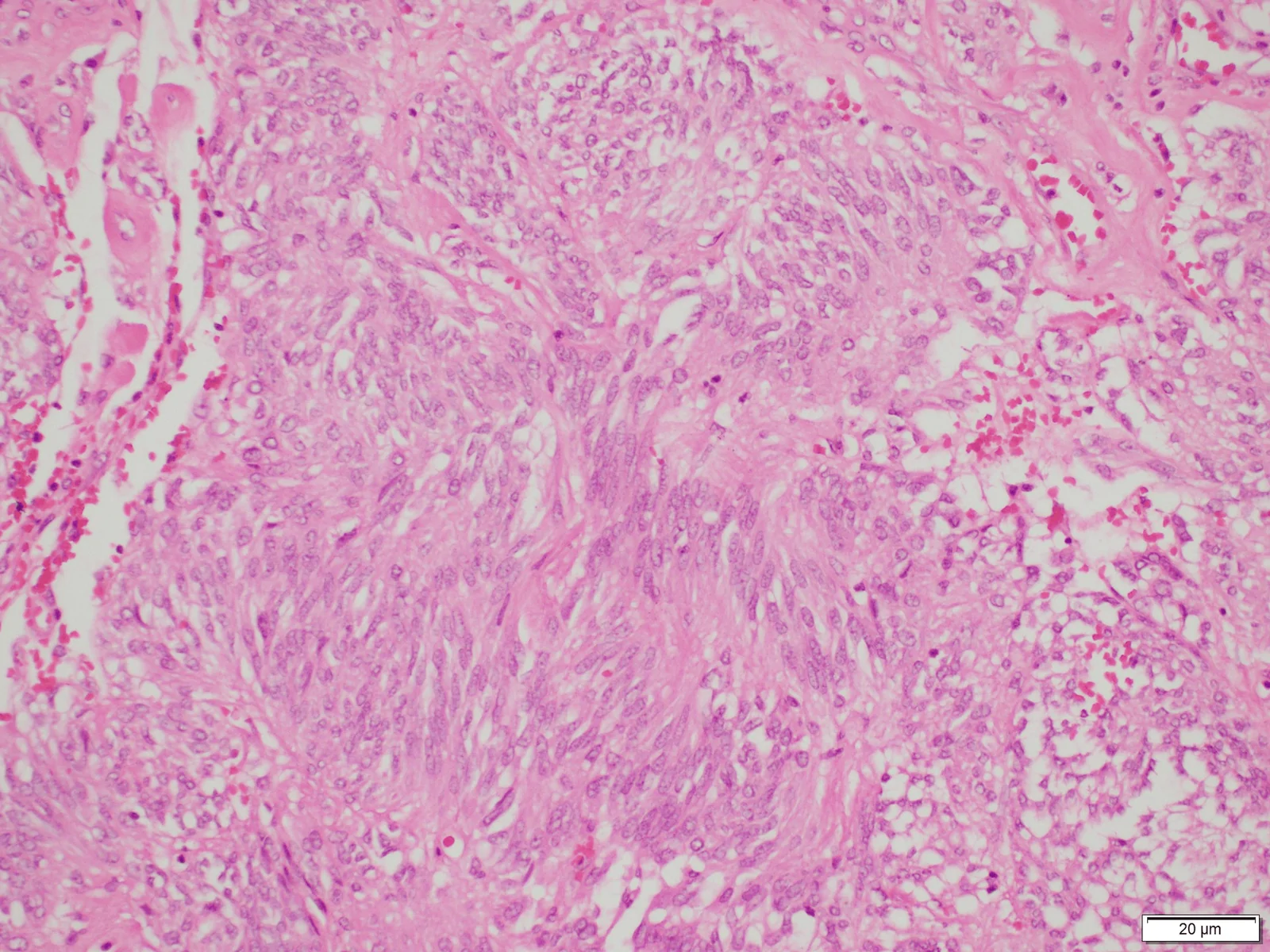
Medium-power view (20×) showing intersecting bundles of bland spindle cells with elongated nuclei, inconspicuous nucleoli, and faintly eosinophilic cytoplasm.

**Figure 6:**
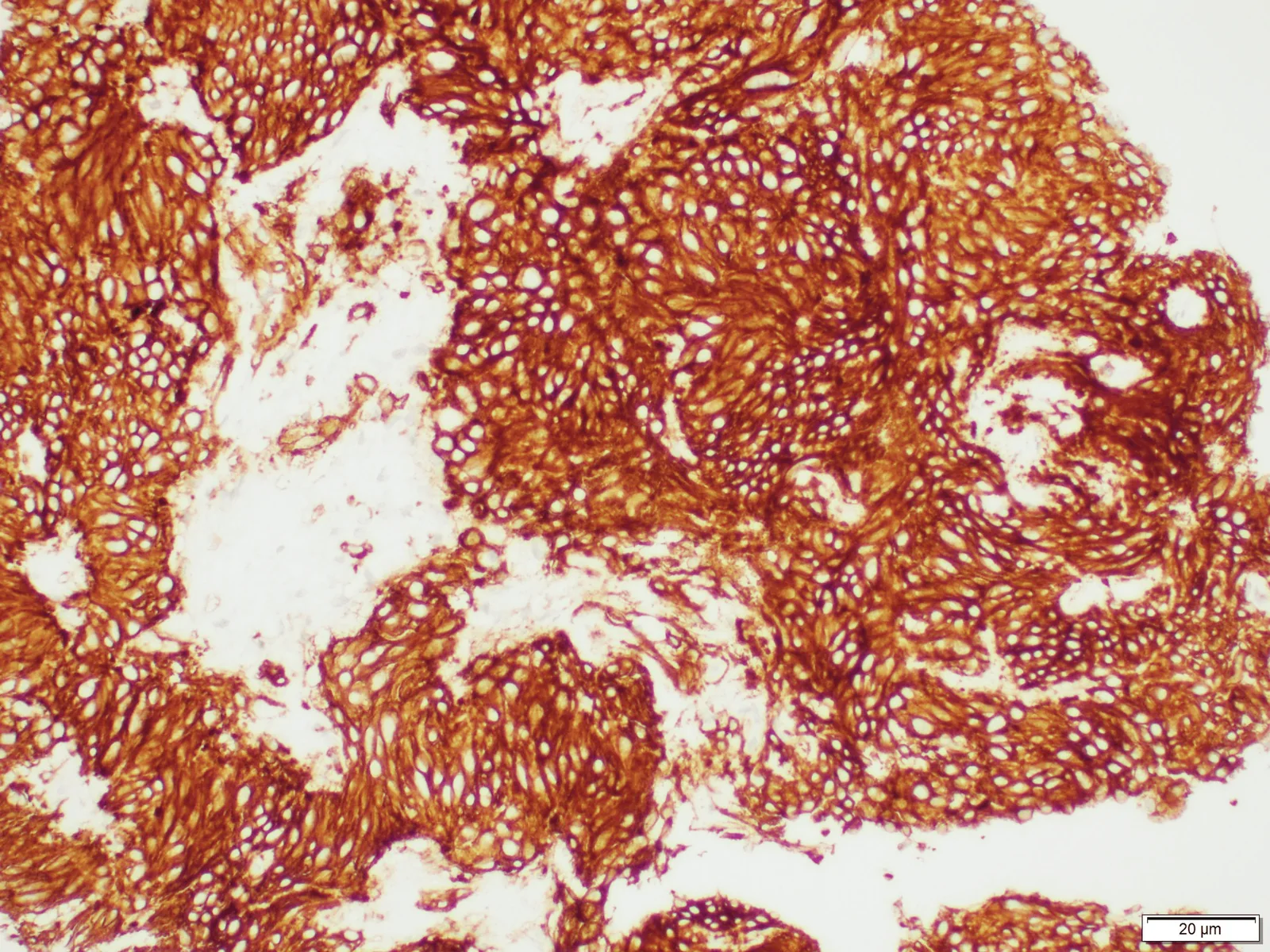
Medium-power view (20×) demonstrating DOG1-positive immunostaining.

**Figure 7:**
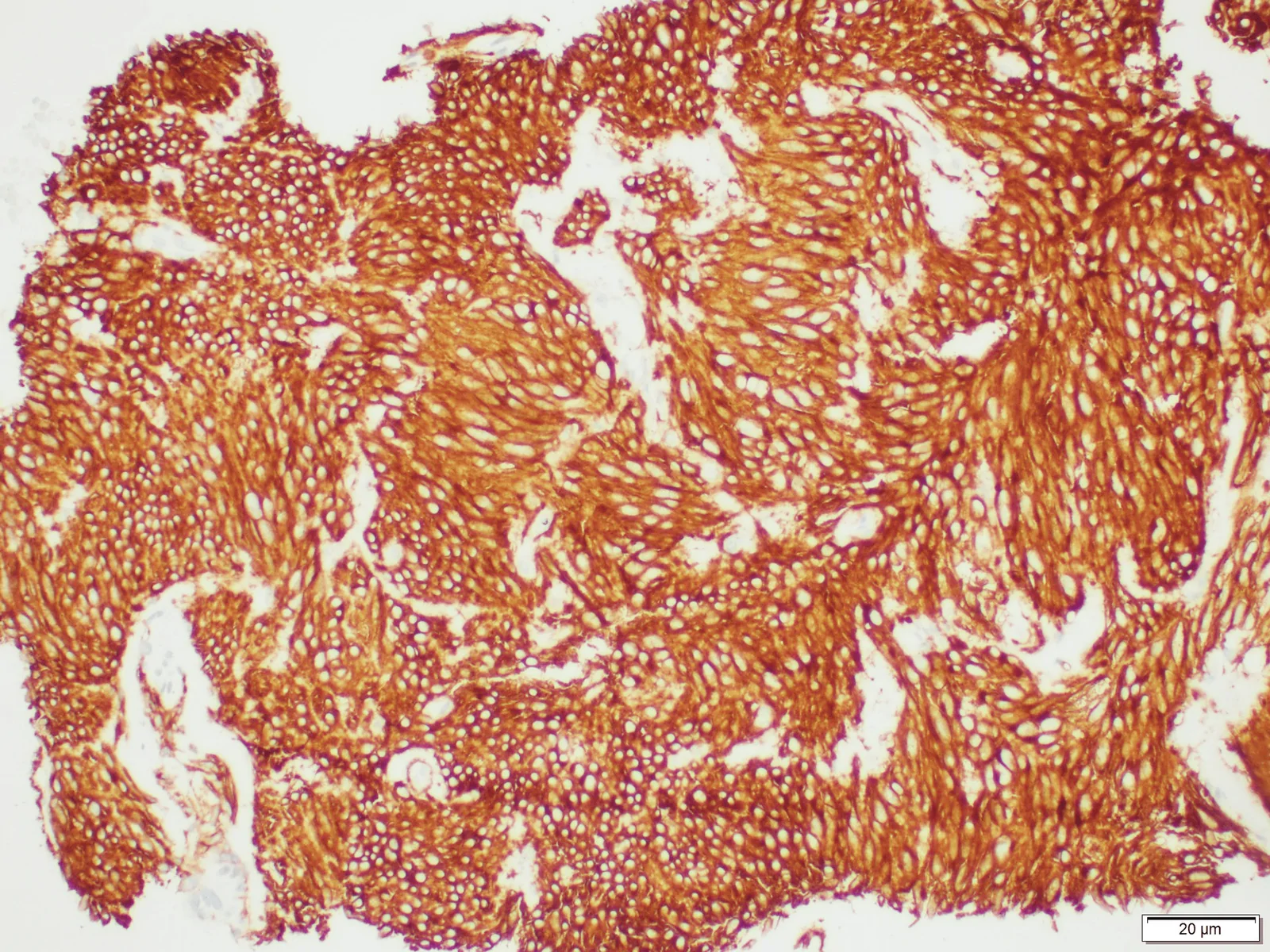
Medium-power view (20×) demonstrating CD117-positive immunostaining.

Following multidisciplinary team discussion, the patient underwent laparoscopic wedge resection. Gross examination demonstrated a gray-white solid tumor with a firm consistency and no evidence of necrosis. Histopathological examination revealed a spindle-cell neoplasm composed of fascicles of bland spindle cells (Figure 4). Higher-power examination demonstrated intersecting bundles of spindle cells with elongated nuclei, inconspicuous nucleoli, and faintly eosinophilic cytoplasm (Figure 5). Immunohistochemical staining was positive for DOG1 and CD117 (Figures 6, 7), supporting the diagnosis of GIST.

The resected specimen measured 6.5 × 5.5 × 4.5 cm. Final histopathological analysis confirmed a mixed-type GIST, G1, low risk, pT3, with a mitotic rate of 3 mitoses per 5 mm². Surgical margins were negative for tumor involvement.

The postoperative course was uneventful. The patient continued pantoprazole therapy and was discharged in stable condition.

## Discussion

Current published literature includes only a limited number of reports describing the coexistence of GISTs and Dieulafoy’s lesions or Dieulafoy-like vascular abnormalities. Several cases have described GISTs presenting with severe GI hemorrhage associated with prominent tumor-related vessels or overlying vascular lesions, suggesting that local vascular alterations may contribute to the bleeding phenotype of some GISTs [2,6-8]. The present case of a 71-year-old male with two episodes of upper GI bleeding separated by seven years adds a distinct temporal dimension to these previously reported associations.

In one reported case, an 86-year-old male presented with melena and was found to have a gastric GIST with an overlying Dieulafoy’s lesion. Endoscopic hemostasis was successfully achieved, followed by surgical resection for definitive treatment [2]. Similarly, a 64-year-old female presented with hemorrhagic shock secondary to a gastric GIST associated with a Dieulafoy-like vascular lesion. Surgical resection confirmed the diagnosis, with bleeding attributed to dilated peritumoral vessels [6]. Another report described a 69-year-old male with severe upper GI bleeding caused by a duodenal GIST associated with a Dieulafoy’s lesion. Endoscopic clipping achieved initial hemostasis before definitive surgical resection was performed [7].

Compared with these previously reported cases, our patient’s clinical course was distinguished by a seven-year interval between the initial diagnosis of a Dieulafoy’s lesion and the subsequent diagnosis of a gastric GIST in the same anatomical region. To our knowledge, this prolonged temporal sequence has not been previously described. Although this observation raises the possibility of an association between localized vascular abnormalities and subsequent tumor development, it does not establish a causal relationship. Unlike previous cases [2,6,7], the prolonged timeline allowed multidisciplinary management, with endoscopic, radiologic, and histopathologic tools guiding diagnosis and treatment.

A possible explanation for this unusual association is that a microscopic or subclinical GIST may have been present during the patient’s initial presentation in 2017 but remained undetectable on endoscopic evaluation. However, this hypothesis remains speculative and cannot be confirmed retrospectively. Alternatively, the Dieulafoy’s lesion and the subsequently diagnosed gastric GIST may represent two independent pathological entities that occurred coincidentally in the same anatomical region. GISTs are well recognized to present with GI hemorrhage secondary to mucosal ulceration, tumor necrosis, or erosion into adjacent vessels, which may complicate differentiation from other vascular causes of upper GI bleeding [4,9]. The relatively indolent clinical course observed in our patient is consistent with the tumor’s gastric location, low histologic grade (G1), and low mitotic rate (3 mitoses/5 mm²), all of which are associated with less aggressive tumor biology [9-11]. Although the resected tumor measured 6.5 cm, these pathological features may explain why it remained clinically silent for several years before presenting as a significant source of bleeding.

The shared need for prompt endoscopic hemostasis and definitive surgical intervention underscores the importance of recognizing this rare association. Similar to previously reported cases, definitive management ultimately required a combination of endoscopic hemostasis and surgical resection, highlighting the importance of a multidisciplinary approach when rare bleeding etiologies coexist [2,6-8]. However, our case highlights the added complexity of recurrent bleeding over time and emphasizes the value of comprehensive follow-up and multidisciplinary collaboration in managing such atypical presentations.

## Conclusions

Recurrent upper GI bleeding requires careful reassessment even when an initial cause has been identified. This case highlights the rare sequential occurrence of Dieulafoy’s lesion and gastric GIST in the same anatomical region. It emphasizes the importance of maintaining diagnostic vigilance and a multidisciplinary approach in patients with recurrent or atypical GI bleeding.
